# Studies Using the Multiphase Optimization Strategy in Digital Mental Health: Scoping Review

**DOI:** 10.2196/98855

**Published:** 2026-11-13

**Authors:** Michaela Kaneva, Diana Esteve Alguacil, Claire Rosalie van Genugten, Annet Kleiboer

**Affiliations:** 1 Department of Clinical, Neuro- and Developmental Psychology, Faculty of Behavioural and Movement Sciences, Vrije Universiteit Amsterdam, De Boelelaan 1105, Amsterdam, 1081 HV, The Netherlands; 2 Amsterdam Public Health Research Institute, Amsterdam, North Holland, The Netherlands; 3 Faculty of Psychology, Universitat de València, València, Spain

**Keywords:** Multiphase Optimization Strategy, MOST, digital mental health, intervention development, optimization, eHealth, factorial design, scalability

## Abstract

**Background:**

Improving the cost-effectiveness and scalability of mental health interventions remains challenging. The Multiphase Optimization Strategy (MOST) framework provides a structured approach for developing, optimizing, and evaluating behavioral interventions by balancing effectiveness with efficiency, economy, and scalability. It does so across 3 phases: preparation (theoretical grounding and component selection), optimization (experimental testing), and evaluation (confirmatory trial). Digital mental health interventions are especially suited to this modular approach, as they enable researchers to easily manipulate components and personalize delivery.

**Objective:**

This study aims to examine how the MOST framework has been applied in the digital mental health field and to provide recommendations for future application.

**Methods:**

Systematic searches were conducted in Embase, CINAHL, Web of Science, PsycInfo, and PubMed. Papers were included if they used the MOST to develop or optimize a digitally delivered mental health intervention. Studies targeting children aged under 12 years, reviews, and theoretical or simulated applications were excluded.

**Results:**

Thirty-seven papers representing 18 research projects met the inclusion criteria. The preparation phase was often incomplete and inconsistently reported, with optimization criteria largely absent and conceptual models often lacking specificity. Eight projects completed at least 1 optimization trial, typically using factorial designs, but progression to intervention refinement or evaluation was limited. Projects conducting multiple optimization rounds were more likely to achieve an optimized intervention.

**Conclusions:**

The MOST is increasingly applied in digital mental health research but often only partially implemented. While factorial designs are widely adopted, critical preparatory elements are frequently missing. To fully realize the MOST’s potential, future research should emphasize comprehensive application across all phases, iterative refinement, and transparent reporting.

## Introduction

### Background

Mental health conditions remain among the most prevalent global health concerns, with current trends indicating that nearly half of the world’s population will have experienced at least one mental disorder by the age of 75 years [1]. Despite the high prevalence, fewer than half of those in need seek professional support [2]. For those who do, access is often limited by long waiting times, high costs, and a shortage of trained professionals [3-7]. Even when treatment is received, many individuals do not experience a meaningful improvement, despite evidence that many psychotherapeutic treatments are generally effective [8-11]. These limitations point to the need for interventions that are more accessible, personalized, efficient, and scalable. Digital mental health interventions (DMHIs) show great promise in overcoming common barriers to care and reducing the treatment gap [12]. For example, they can lower delivery costs, increase accessibility in remote regions, and enable personalization, a potential amplified by rapid advances in AI. DMHIs take on various forms; they can complement the clinicians’ work or function as fully autonomous, unguided formats, including web and smartphone-based modalities [13]. Some smartphone apps are now formally recognized as medical tools that can be prescribed and reimbursed by health care providers in several countries such as the Netherlands, Germany, and the United Kingdom [14,15]. Importantly, the potential of digital tools extends beyond treatment. As unguided formats are usually low-intensity, they are well suited for prevention and health promotion, for example, by offering interventions that improve coping skills and resilience [16]. In this context, prevention may involve targeting risk factors or strengthening protective factors and mental well-being. Despite their potential, DMHIs face challenges similar to face-to-face interventions. While they are generally effective, treatment response varies across individuals, and improving their overall (cost) effectiveness over time remains difficult. To enhance effectiveness, it is important to precisely understand *how* these interventions achieve their effects. Randomized controlled trials (RCTs), considered the gold standard for evaluating interventions, assess the global effectiveness of an intervention against a control condition, but they do not allow researchers to isolate which components are responsible for change, how these elements interact, or whether they operate through the hypothesized mechanisms [17,18]. This way, it is difficult not only to improve interventions over time but also to identify ineffective or even potentially harmful components, making them less effective but also less efficient and economical. Finally, the interventions that are tested within an RCT are often not scalable because they are too complex, resource-intensive, and insufficiently tailored to local contexts and policies [19,20]. Such interventions are often altered postevaluation. However, because the exact mechanisms through which interventions work are not certain, decisions about how to modify intervention packages to fit real-world demands rely on theoretical assumptions and practical considerations rather than empirical data. This risks perturbing active ingredients and the underlying working mechanisms. At the same time, retaining components that contribute to effectiveness may come at the cost of greater intervention length, participant burden, or resource use, potentially affecting adherence and scalability. These trade-offs are difficult to evaluate post hoc and should ideally be considered explicitly prior to evaluation. The Multiphase Optimization Strategy (MOST) [21] is a framework designed to tackle the limitations of the traditional intervention development and evaluation. Inspired by engineering and industry, it aims to prevent suboptimal allocation of resources during optimization by ensuring that intervention components are tested efficiently and purposefully, rather than through overly resource-intensive approaches. The MOST is guided by 2 core principles: continual optimization, which emphasizes iterative intervention refinement over time, and resource management, which prioritizes the efficient use of available resources across both intervention development and testing. By experimentally assessing individual and combined intervention components and applying explicit implementation constraints, the MOST balances effectiveness with the need for efficiency, economy, and scalability. In doing so, it provides a systematic and transparent approach to intervention optimization, illustrating that effectiveness, efficiency, affordability, and scalability are interrelated and can be targeted simultaneously. The MOST framework can be applied both to the development of new interventions and to the optimization of existing ones. It is structured into 3 phases: preparation, optimization, and evaluation [22].

### Preparation Phase

The preparation phase lays the groundwork for optimization. The *conceptual model* is considered the “engine” that drives the intervention [23], that is, its development, its translation into a trial design, and the interpretation of the trial results. A strong model clearly specifies the relations among the key constructs, depicting the causal pathways through which the intervention components are expected to influence the intervention target (ie, the “primary” or “ultimate” outcome of interest). These causal pathways must include mechanisms of change. The mechanisms of change must be operationalized (and measured) as proximal outcomes expected to affect the “ultimate” outcome of interest, which often occurs later, thus sometimes also called a “distal” outcome. For example, in an intervention targeting insomnia (distal outcome), components, such as physical activity, light control, and journaling (intervention components), may reduce insomnia through lessening stress, improving the circadian rhythm, and reducing worrying (mechanisms of change). Ideally, each intervention component should target a specific mechanism of change to strengthen the interpretation of the optimization trial results and thereby facilitate the decision-making on which combination of components to retain. The intervention can be developed using the literature, but it is also beneficial to incorporate lived experience through co-design and input from end users and stakeholders [24]. Additionally, it can be beneficial to consider *effect modification*, that is, to assess person-specific variables which may impact the effectiveness of components (such as presence of comorbidity or demographic characteristics). This can help identify which components work best for whom. Next, an *optimization criterion* is defined. The optimization criterion specifies a (set of) key implementation constraint(s), within which the desired outcome (such as an effective intervention) should be achieved. This involves setting limits on resources so that effectiveness can be balanced against the efficiency, economy, and scalability. An example would be a behavioral intervention for lifestyle change that produces clinically significant change (outcome) while not exceeding 10 sessions (constraint). In a hypothetical scenario of unlimited resources, an “all active components criterion” can be used, allowing the inclusion of all components that demonstrate an effect. Finally, before proceeding to the optimization phase, the candidate components are *pilot tested* for feasibility, usability, and acceptability to ensure that each element can be delivered as intended.

### Optimization Phase

For the *optimization phase*, an experimental method is used to determine which (*candidate) components* [1] produce meaningful effects, either independently or in combination. The choice of experimental design depends on the intervention design. For “fixed interventions,” where all participants in the same condition receive the same content, a factorial design is used. For “adaptive interventions,” where content is personalized according to participant input, different approaches are suitable such as a sequential multiple assignment randomized trial (SMART) or a microrandomized trial. Based on the results of the optimization trial, it is decided which (combination of) components to retain in the optimized intervention package. This *decision-making process* follows three steps: (1) identifying an effect size that a component should demonstrate alone or in combination to be considered important (“effect criteria”), (2) splitting components into those that qualify for further consideration (“screened-in set”) and those who do not (“screened-out set”) according to the effect criteria, and (3) selecting the best combination of screened-in components that meet the optimization criterion. If the trial does not produce interpretable effects, meaning that there is no difference in components on their effectiveness or other type of resource-intensiveness, a return to the preparation phase is necessary. Accordingly, multiple optimization rounds may be needed, which includes returning to the preparation phase for revision. However, even successfully optimized interventions can be further optimized in the future, in line with MOST’s continual optimization principle.

### Evaluation Phase

Once an optimized intervention has been achieved, it can be evaluated in a conventional RCT against a control condition. If effectiveness is demonstrated, the intervention is ready for large-scale implementation, having already been optimized with respect to key resource constraints in the preceding stage.

### Research Objectives

Digital (complex) interventions are particularly well suited to the MOST framework due to their capacity to precisely manipulate therapeutic components and the extensive opportunities for personalization. However, the extent and manner in which theoretical frameworks are applied differ across studies, highlighting the need for a clearer overview of current practice [25,26]. Therefore, the aim of this scoping review is to examine how the MOST has been applied to the development, optimization, and evaluation of DMHIs and to provide recommendations for future application. A scoping review methodology was selected instead of a systematic review because the scope, characteristics, and extent of the evidence were not yet well established and expected to be heterogeneous, making a broad mapping approach more appropriate for a broad research question than a narrowly focused approach [27].

## Methods

### Search: Eligibility Criteria, Databases Consulted, and Search Strategy

This review was conducted following the PRISMA-ScR (Preferred Reporting Items for Systematic Reviews and Meta-Analyses Extension for Scoping Reviews) guidelines. A completed checklist indicating where every item was reported can be found in Checklist 1. The review was not preregistered because the approach to organizing and synthesizing the evidence was refined iteratively as the scope and heterogeneity of the literature became clear. A systematic search was carried out in July 2024 (and updated in August 2026) in the databases Embase, CINAHL, Web of Science, PsycInfo, and PubMed. In all of them, abstracts, titles, and keywords were initially screened for the search terms: “multi-phase optimization strategy” OR “multiphase optimization strategy” OR “multi-phase optimization strategy” OR “most framework” (Multimedia Appendix 1). These search terms were derived from the systematic review of Landoll et al [28] of the preparation phase of the MOST framework. The current review extends this scope by additionally examining the optimization and evaluation phases, while narrowing the focus to DMHIs, enabling a field-specific synthesis of MOST implementation. The eligibility criteria consisted of (1) the paper used the MOST to develop or optimize an intervention, or the paper was part of a research project that did so; (2) the intervention targeted a mental health problem; and (3) the intervention was partially or fully delivered digitally. Interventions targeting children (aged <12 years) were excluded as well as theoretical examinations of the MOST framework, review papers, hypothetical applications to a new field, and simulated experiments. Preprints were eligible for inclusion.

### Selection Process

After removing duplicates, 2 reviewers (AK, DEA) first independently screened the title and abstract of all search results to determine if they met the eligibility criteria. Following that, 2 reviewers (MK, CRvG) conducted full-text screening to further determine inclusion. Any conflicts or disagreements were resolved by jointly discussing the relevant papers and analyzing their adjustment to the eligibility criteria (MK, DEA, CRvG, AK). Additionally, once projects were selected for inclusion, supplementary forward and backward searches were conducted (MK) to identify papers within the projects that might have been missed in the initial search due to not referencing the MOST framework. Such cases typically occurred in publications preceding or following the optimization phase, such as intervention development and pilot testing or RCTs. This step was aimed to capture information relevant to the review, such as the theoretical background of the intervention, an existing pilot trial, or outcomes from an RCT that may have occurred either prior to a MOST preparation and optimization phase or following a MOST optimization phase.

### Data Extraction and Synthesis

Once the set of included papers was established, data were extracted using customized Excel spreadsheets (Multimedia Appendix 2). These spreadsheets collected extensive data on each paper. First, general information about the study was extracted (authors, publication year, location, type of article [empirical, protocol paper], part of which research project, etc), followed by general information about the MOST (what phase[s] did the paper refer to) and then detailed information about each of the MOST phases, including the activities recommended per phase. The unit of analysis was the underlying research project rather than the individual papers. Accordingly, multiple papers within a single research project were considered to represent one study, for instance, separate publications of the preparation and optimization phases, or a protocol paper describing an optimization trial and a subsequent paper reporting the trial results.

Additionally, a risk of bias assessment was conducted for the completed (MOST) trials. Two reviewers independently conducted the assessments based on the 5 domains of Cochrane’s RoB 2 tool [29], with disagreements resolved through discussion. The outcomes for each of the five criteria are provided in Multimedia Appendix 3, and detailed ratings are available on request.

## Results

### Study Selection

Thirty-seven papers met the inclusion criteria, comprising 11 protocol papers and 26 empirical papers. These papers corresponded to 18 distinct research projects (see Figure 1 for the inclusion process).

**Figure 1.**
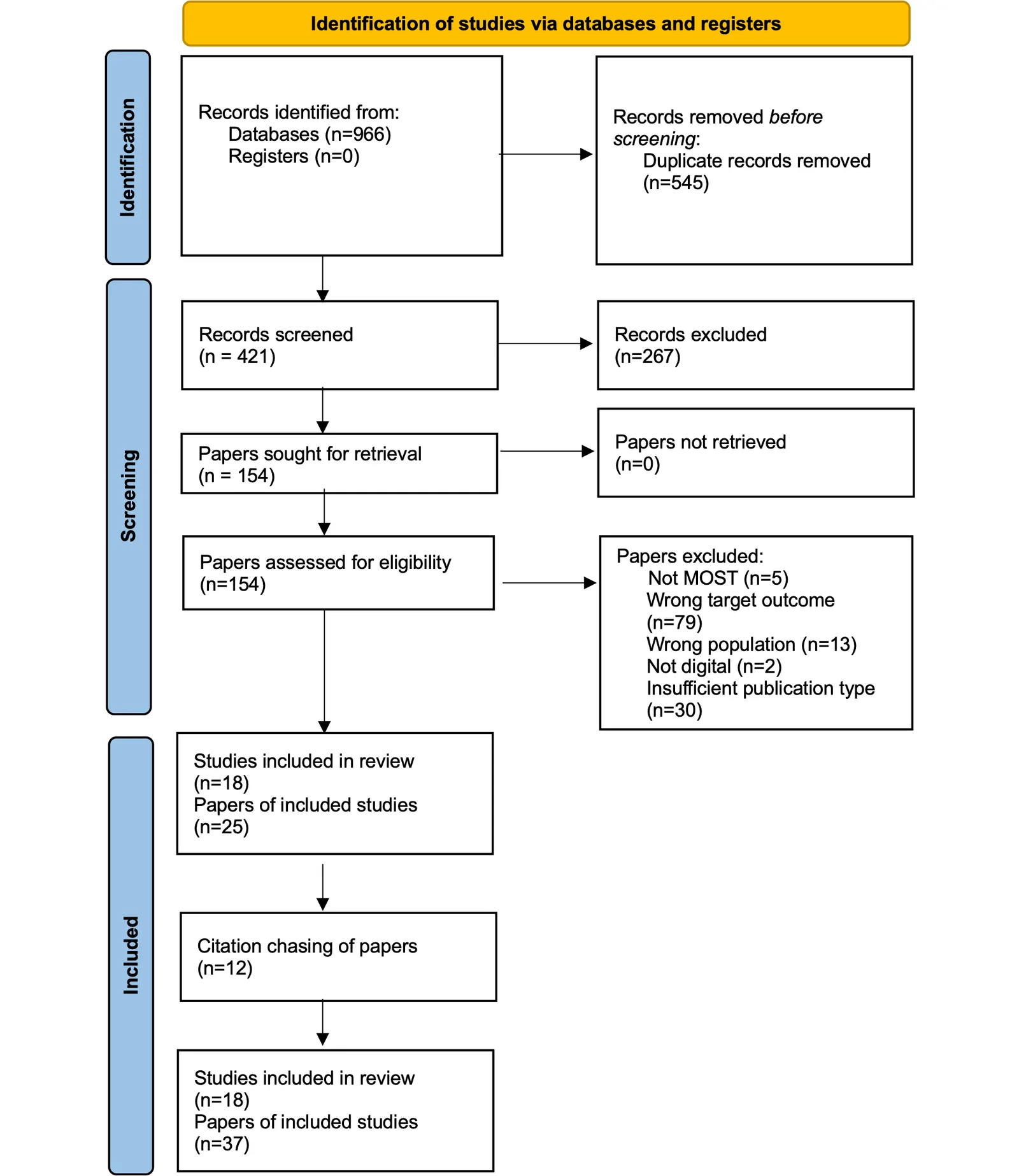
Study inclusion. MOST: Multiphase Optimization Strategy.

### General Characteristics of the Included Studies and Their Interventions

Half of the projects were conducted in the United States (n=9), followed by the United Kingdom (n=3), Japan (n=2) and China (n=2), Canada (n=1), and Singapore (n=1). All papers were published between 2013 and 2026; the first paper, which explicitly mentioned MOST, was published in 2016 [30]. The most common primary outcome was depressive symptoms measured with the 9-item Patient Health Questionnaire. Table 1 shows an overview of the included studies and the associated papers, as well as the investigated interventions’ content and delivery. Seven interventions were entirely self-guided, one was adaptive, and one had a component with an adaptive element.

**Table 1.** Overview of the intervention characteristics and associated papers per research project.

Research project	Intervention						Papers
	Population	Outcome	Components	Design	Delivery	Sample size	
BioF^a^	Adults with work-related stress	Work-related stress	Daily resting HRV^b^ biofeedback (1), momentary HRV biofeedback (2), behavioral initiation coaching (3), practice with feedback coaching (4)	Fixed	A smartphone app for (1) and (2), a video conferencing tool or via telephone for (3) and (4). Additionally, a website was available with behavior change components for all participants	16	A pilot trial [31]
eMindfulness Mode (eMM)	Young people	Risk factors for disordered eating	Decentered awareness and acceptance of present-moment experience (1), emotion regulation (2), self-compassion (3)	Fixed	Website	80	Intervention development [32]; a pilot trial [33]
Practice Experiences for School Reintegration (PrESR)	Adolescents hospitalized for suicide-related crises	Suicide-related behavior after hospitalization	Affect regulation (1), problem-solving (2), cognitive restructuring (3)	Fixed	VR^c^	42	Intervention development pilot trial [34,35]
LvL UP (LUP)	General population	Mental well-being	Coaching sessions, behavioral tasks, breathing exercises, journaling—all components enhanced by storytelling, gamification, just-in-time notifications, peer support (LvL UP Buddy) (1); MI^d^ with human support (2)	Adaptive	Smartphone app, including agent-delivered coaching; motivational interviewing with a trained research assistant for nonresponders	120 for PT^e^, 650 for OT^f^	Intervention development [24]; optimization trial protocol [36]; a pilot trial [37]
CATCH-IT^g^ (CIT)	Adolescents with subsyndromal symptoms of depression	Depression prevention	Behavioral activation (1), CBT^h^ adolescent component (2), interpersonal therapy adolescent component (3), parent program (4)	Fixed	The components are delivered via a website. Additionally, for the purposes of engagement, the intervention also includes MI telecoaching	400	Optimization trial protocol [38]
Healthy Minds Program (HMP)	Sexual and gender minorities of color	Discrimination-related stress and well-being	Mindfulness-based behavioral intervention: awareness (1), connection (2), purpose (3)	Fixed	Smartphone app	80	Optimization trial protocol [39]; pilot trial [40]
REACH+^i^ (RCH)	Adults with anorexia nervosa who have achieved weight restoration	Relapse of anorexia nervosa	Behavior (1), cognition (2), motivation (3), consolidation (4), food monitoring (5)	Fixed	The candidate components are delivered via telehealth sessions with a therapist; additionally, a web-based platform is available between sessions, with personalized content according to condition assignment	60	Optimization trial protocol [41]
TEEM^j^ (TEM)	In-patients at a psychiatric hospital	Postdischarge suicide risk	Routine tracker (1), mind care (2), social connectivity (3), achievement and rewards (4)	Fixed	Gamified smartphone app	320	Intervention development and optimization trial protocol [42]
EMPOWER^k^ (EMW)	Adolescent and young adult cancer survivors	Mental well-being	Gratitude (1), mindfulness (2), positive reappraisal (3), personal strengths goal setting (4), acts of kindness (5)	Fixed	Website/platform with a discussion tab	352	Optimization trial protocol [43]
BSI^a^ CBT-I^l^	Adult patients with cancer with poor sleep quality	Sleep difficulties and insomnia	Sleep restriction (1), stimulus control (2), bright light (3)	Fixed	Introductory session via videoconferencing/in person, followed by email administration of the candidate intervention components	13 for PT, 80 for OT	Optimization trial protocol [44]; pilot trial [45]
LIFT	Cardiorespiratory failure survivors with elevated distress	Distress	Mindfulness-based intervention, including rationale for mindfulness, guided meditation practice, and additional tips for mindfulness in daily life; variations: therapist vs application only delivery (1), high vs low dose meditation exercise (2), initiating intervention (therapist vs application) (3)	Fixed/adaptive	Webpages and smartphone apps; therapists deliver the intervention via telephone instead of the app	80 PT, 247 for OT	Intervention development [46]; optimization trial protocol [47]; pilot trial [48]; factorial trial [49]; process evaluation [50]
Healthy Campus Trial (HCT)	University students with elevated stress	Depression	Psychoeducation (1), self-monitoring (2), behavioral activation (3), cognitive restructuring (4), assertiveness training (5), problem-solving (6)	Fixed	Smartphone app	1088	Optimization trial protocol [51]; optimization factorial trial results [52]
IMPROVE^m^ (IMV)	Adults with depression	Depression	Absorption training (1), activity scheduling (2), concreteness training (3), functional analysis (4), relaxation (5), self-compassion training (6), thought challenging (7)	Fixed	Internet platform and contact with therapists online	767	Optimization Trial protocol [30]; trial results [53]
J-SUPPORT Study (JSP)	Patients with cancer	Depression	Psychoeducation (1), behavioral activation (2), assertiveness training (3), problem-solving (4)	Fixed	Smartphone app	359	Evaluation of the intervention as a package (RCT)^n^ prior MOST^o^ [54] Optimization trial protocol [55] Optimization trial results [56]
FoRtitude (FoR)	People with a history of breast cancer	Fear of recurrence	Core component: psychoeducation; candidate components: relaxation (1), cognitive restructuring (2), worry practice (3), motivational interviewing (4)	Fixed	eHealth site (1), (2), and (3), telecoaching (4)	196	Optimization trial results [57]
MID^a,p^	Adult caregivers with symptoms of depression	Distress	Self-care skills (1), behavioral problem management (2), behavioral activation (3), mindfulness-based intervention (4), and support group (5)	Fixed	All components were delivered via telephone or videoconferencing by trained research assistants	250	Optimization trial results [58]
myPlaybook (PlyB)	College athletes	Substance use	Harm prevention (1), norms module (2), expectancies module (3)	Fixed	Web-based platform	Trial 1: 3244; trial 2: 2837; trial 3: 2193	Evaluation of the intervention as a package (RCT) prior MOST intervention optimization [59]; Iterative optimization trials—results and intervention refinement [60]
Drink Less (DrL)	Adults who drink excessively	Alcohol consumption	Normative Feedback (1), Cognitive Bias Retraining (2), Self-monitoring and Feedback (3), Action Planning (4), Identity Change (5)	Fixed	Smartphone app	672 for OT, 5602 for RCT	Intervention development [61]; optimization trial results [62]; Returning to preparation phase for refinement [63] process evaluation [64]; RCT of intervention against control condition [65]

a This project did not have an intervention or a trial name.

b HRV: Heart rate variability.

c VR: virtual reality.

d MI: motivational interviewing.

e PT: pilot trial.

f OT: optimization trial.

g CATCH-IT: Competent Adulthood Transition with Cognitive-Behavioral Humanistic and Interpersonal Therapy.

h CBT: cognitive behavioral therapy.

i REACH+: Relapse Prevention and Changing Habits.

j TEEM: Tailored Evidence-­Based Enhancements in Mental Health.

k EMPOWER: Enhancing Management of Psychological Outcomes With Emotion Regulation.

l CBT-I: cognitive behavioral therapy for insomnia.

m IMPROVE: implementing multifactorial psychotherapy research in online virtual environments.

n RCT: randomized controlled trials.

o MOST: Multiphase Optimization Strategy.

p MID: multicomponent intervention for distressed informal caregivers of people with dementia.

### Detailed Results per MOST Phase

#### Overview

Out of the 18 projects, half focused on the optimization phase only (n=9), 3 on the preparation phase, and 5 on both. Additionally, 1 project covered all phases. Table 2 depicts an overview of the activity completion per phase.

**Table 2.** Overview per Multiphase Optimization Strategy (MOST) phase per study.

Phases and activities	Project progression across MOST phases																	
	Phase 1 (intervention development and pilot trial only)			Optimization trial planned (protocol paper only)							Optimization trial completed (results available)							Evaluation completed (RCT^a^)
	BioF^b^	eMM^c^	PrESR^d^	LUP^e^	CIT^f^	HMP^g^	RCH^h^	TEM^i^	EMW^j^	BSI^k^	LIFT	HCT^l^	IMV^m^	JSP^n^	FoR^o^	MID^p^	PlyB^q^	DrL^r^
Phase 1																		
Components identified	✓	✓	✓	✓	✓	✓	✓	✓	✓	✓	✓	✓	✓	✓	✓	✓	✓	✓
Conceptual model	✓			✓		✓	✓			✓	✓					✓	✓	✓
Optimization criterion																	✓	
Pilot trial	✓	✓	✓	✓		✓			✓	✓	✓	✓	✓		✓			✓
Phase 2																		
Experimental design	FT^s^	FT	FT	SMART^t^, PE^u^	FT	FT	FT	FT	FT	FT	FT PE	FT	FT	FT	FT	FT	Three sequential FTs	FT PE
Effect criterion specified (decision-making step 1)							✓	✓			✓						✓	
Screen-in/Screen out (decision-making step 2)											✓	NA^v^	NR^w^	NR	NA	NR	✓	
Screened-in components that meet the optimization criterion (decision-making step 3)											NA	NA	NA	NA	NA	NA	✓	N/A
Revision of intervention after an optimization trial											NA	NR	NR	NR	NR	NR	Changes in the conceptual model, the outcome, and the effect	3 new modules added, 1 removed
Phase 3																		
RCT																		✓

a RCT: randomized controlled trials.

b Determining the right levels of health coaching and heart rate variability biofeedback in a workplace behavior change intervention: Multiphase Optimization Strategy preparation study.

c eMM: eMindfulness Mode.

d PrESR: Practice Experiences for School Reintegration.

e LUP: LvL UP.

f CIT: CATCH-IT: Competent Adulthood Transition with Cognitive-Behavioral Humanistic and Interpersonal Therapy.

g HMP: Healthy Minds Program.

h RCH=REACH+: Relapse Prevention and Changing Habits.

i TEM=TEEM: Tailored Evidence-­Based Enhancements in Mental Health.

j EMW=EMPOWER: Enhancing Management of Psychological Outcomes With Emotion Regulation.

k BSI: Optimizing a Behavioral Sleep Intervention for Gynecologic Cancer Survivors.

l HCT: Healthy Campus Trial.

m IMV=IMPROVE: Implementing multifactorial psychotherapy research in online virtual environments.

n JSP: J-SUPPORT Study.

o FoR: FoRtitude.

p MID: multicomponent intervention for distressed informal caregivers of people with dementia.

q PlyB: myPlaybook.

r DrL: DrinkLess.

s FT: factorial trial.

t SMART: sequential multiple assignment randomized trial.

u PE: process evaluation.

v NA: not applicable.

w NR: not reported.

#### Preparation Phase

##### Candidate Components

All studies identified candidate components to be tested. These components were either directly adapted from existing interventions or derived through a synthesis of the literature and/or collection of qualitative data from end users and stakeholders.

##### Conceptual Model

Eleven studies mentioned either a “conceptual model” [24,31,35,41,45,47,60,62] or a similar structure (ie, a “conceptual framework” [39], “theoretical design” [38], or a “logic model” [32]). Of 12 specified mechanisms of change [24,30,33,34,38,40,41,43,45,58,60,65], 7 had a level of differentiation when it comes to which modules target which mediators [30,40,41,45,58,60,66], and 5 operationalized those into measurable outcomes [30,41,43,58,60]. None of the studies prespecified expectation in terms of moderation (such that concrete modules interact in a concrete way), but all that conducted (or planned) an optimization trial set out to test moderation. Five studies explored person-specific variables for effect modification [38,43,51,57,66].

##### Optimization Criterion

Only 1 study explicitly reported an optimization criterion [60]: “the largest effect size achievable after 2 optimization trials.” Nonetheless, a qualitative, exploratory analysis of the stated objectives indicated that optimization criteria were often deducible. Concretely, an “all-active-components” criterion was implied in 10 studies; this was evident either where an optimized intervention was defined as 1 with the most efficacious combination of components [39,42] or where factorial experimentation was framed as a means to identify the most effective “combination” of components, to determine which components are “active,” or to examine main and interaction effects of the candidate components [41,43,44,51,53,56-58]. In addition, 2 further studies articulated a resource-oriented trade-off without fully operationalizing this quantitatively. One study (*CIT*) mentioned an objective to identify an intervention component, which is “equally efficacious as a combination of other components while being more tolerable and scalable” [38]. Another study (*LVL-UP*) aimed to identify the “most cost-effective condition” [36], implying a trade-off between cost and effectiveness in selecting components as well.

##### Pilot Trial

Twelve studies [30,31,33,34,37,40,43,45,48,51,57,61] conducted a pilot trial, that is, a study prior to the optimization trial aimed at assessing if the candidate components could be delivered as intended, were understandable to participants, and appeared safe prior to proceeding to the optimization trial. Notably, the reporting terminology for this trial was inconsistent: 4 used a different term (eg, “feasibility trial”) for designs that serve the same purpose as a MOST pilot trial [30,33,44,61], and 1 referred to a “pilot trial” to describe what was substantially an optimization trial [39].

### Optimization Phase

#### Optimization Trial: Experimental Design and Results

Eight projects conducted the optimization phase, all implementing a factorial design [49,52,53,56-58,60,62]. Six of those ran a single optimization trial [49,52,53,56-58], whereas 2 undertook multiple rounds of optimization [60,63]. One intervention (*LIFT*) had a significant main effect immediately postintervention (and a significant interaction at 3-month follow-up), claiming an “optimized” version of the intervention [49]. For the rest, there were no superiority effects of candidate components on the outcomes of interest immediately postintervention [52,53,56,57], while one study had significant interaction effects [62]. At follow-up, 1 study showed a small main effect and 2 interaction effects [53], and another study found main and interaction effects across conditions that differed depending on the outcome assessed (proximal outcomes) [58] measured at 6- and 12-month follow-up only.

#### Decision-Making Following Optimization Trial

##### Effect Criteria

Four studies reported an effect criterion for a component to be in the screened-in or screened-out set (sizes ranging between *d*=0.2 and *d*=0.35) [41,42,47,60]. Additionally, 4 other studies specified as part of the power calculation an expected effect per component ranging between *d*=0.2 and *d*=0.5 [30,43,56,57], thus indicating an expected effect size even if not formulating this as a criterion.

##### Revision

Two interventions underwent revision after the optimization trial as these did not produce results that could be used for optimization due to lack of superiority of any experimental condition. In *My Playbook* [60], after 3 successive optimization trials, the revised package combined modules that met the predefined effect-size criterion, whereas *Drink Less* was refined after 1 optimization trial using a 3-phase mixed-methods approach and then progressed to the evaluation phase [22,65]. By contrast, the only intervention that showed multiple significant effects in the first optimization trial [58] did not report subsequent decision-making regarding the configuration of an optimized intervention package. It is possible that, given that this study focused on multiple proximal outcomes instead of a single distal outcome, determining the best-performing condition was challenging.

##### Risk of Bias

Overall, the completed trials showed low risk of bias. The most common concerns were related to domain 5 (selective outcome reporting), while domains 2 (deviations from the intended protocol) and 4 (appropriate measurement of the outcome) were marked as low risk for all studies (Multimedia Appendix 3).

### Evaluation Phase

One study (*Drink Less*) tested the previously MOST-optimized intervention as a package within a 2-arm parallel group, double-blind, RCT against usual digital care, thus against an effective comparator [65]. At 6 months, the prespecified primary analysis did not demonstrate a statistically significant between-group difference in weekly alcohol consumption; a sensitivity analysis revealed a significant effect on the outcome of interest in favor of *Drink Less*, concretely a 2.00-unit greater weekly reduction of self-reported weekly alcohol consumption.

## Discussion

This scoping review explored how the MOST framework has been applied to the development, optimization, and evaluation of DMHIs. A key strength observed across studies was the careful selection of candidate components and the use of factorial designs to efficiently examine their unique and combined effects. However, only 1 study explicitly defined an optimization criterion, with optimization logic in other studies often remaining implicit. In addition, although first-round optimization trials frequently did not identify components with superior effects, only a small number of studies pursued further rounds of optimization, even though studies that did so eventually achieved an optimized intervention [60,65]. The lack of significant results followed by a limited progression beyond a single optimization trial may partly reflect commonly observed gaps in the preparation phase, particularly limited specification and operationalization of the conceptual model and a lack of clearly articulated resource constraints. Together, these gaps may challenge the intervention development, the assessment of its effectiveness, and information on how to revise. Thus, while researchers are increasingly adopting the factorial trial as a helpful tool for optimization, there is still room to align more closely with broader MOST optimization aims. Given the widespread reliance on the factorial design in combination with an all-active-components criterion without constraints on other resources, it is useful to consider why this approach may be compelling. By identifying ineffective or harmful components, factorial designs appear to support optimization through component removal *alone*, improving effectiveness, efficiency, and economy without further resource constraints. However, the findings of this review indicate that such scenarios remain relatively uncommon. Across studies, first-round optimization trials rarely demonstrated clear superiority of specific components, and in the absence of explicit optimization criteria beyond effectiveness, opportunities to select less resource-intensive configurations can be missed. Thus, greater attention is needed to the fact that optimization does not necessarily imply maximizing effectiveness alone, but trade-offs between effectiveness and factors such as intervention burden, duration, cost, or scalability can be considered within an optimization criterion. In line with this observation, recent work on the evolution of the MOST, by the developer of the MOST herself, Prof Dr Linda Collins, highlights a shift toward framing optimization not solely as the removal of inactive or harmful components but as the systematic evaluation of whether each component’s contribution warrants its associated resource use across both intervention delivery and testing [22]. Overall, the value of explicitly specifying optimization and effect criteria lies less in procedural completeness than in helping researchers make decisions at each step and moving forward optimization. When this element is not fully specified (Table 2), defining what constitutes an important effect (step 1 of the decision-making) becomes difficult, which in turn complicates screening components in or out (step 2) and selecting among screened-in components under an optimization criterion (step 3). Furthermore, without a conceptual model to guide which components are tested in relation to which underlying mechanisms, returning to the preparation phase may become difficult as action points regarding refinement and restructuring of the intervention are less obvious due to missing information on the causal chain linking components to mechanisms of change and distal effects. Notably, a word of caution is warranted when drawing theoretical conclusions from factorial design results—null effects should not be interpreted as evidence that interventions operate primarily through shared or common mechanisms as discussed within “the dodo bird verdict” debate. Rather, such findings may reflect overlapping content and spillover between components [60]. In this sense, the potential of MOST may lie less in uncovering an underlying “true” intervention structure than in challenging researchers to explicitly construct and operationalize one. Finally, the review showed that personalization efforts were relatively limited, with effect modification and adaptive interventions rarely examined. Within MOST, both tailoring strategies were originally positioned as less central, with detailed modeling of effect modification in the conceptual model sometimes discouraged because it was thought to risk distracting from specifying core mechanisms of change [67]. In DMHIs, however, these dimensions are likely to become increasingly important, as real-time data and AI-driven decision rules make individual-level adaptation feasible. A recent review highlights the importance of personal characteristics for engagement with DMHIs, showing that engagement differs systematically by factors such as mental health status and technological literacy, and suggesting that tailoring content to these characteristics offers a promising avenue for customization [67]. Effect modification pertains to for which subgroups components work best, for example, whether effects for a given component differ between participants with more severe symptoms, longer episode duration, or specific comorbidities. Examining such differences can identify components that may be particularly beneficial for individuals with specific clinical characteristics, even when their average effect is small. Failing to account for variability in responses to the various components may obscure the results of the general effectiveness of modules and in some cases lead to the removal of components that are beneficial for particular groups. Adaptive interventions focus on how intervention delivery can be tailored to the individual over time, based on ongoing input such as self-report, usage patterns, or contextual data. SMART designs used to assess adaptive interventions are highly complex and can be challenging to plan and evaluate, which may partly explain their limited use. Additionally, some distal outcomes may be particularly challenging for optimization. For example, in prevention, demonstrating reductions in the incidence of a full-blown disorder can require months or years of follow-up, making rapid iterative optimization based on the ultimate outcome difficult. In such cases, other (proximal) outcomes, such as established risk factors or precursors of disorders, may provide more readily assessable targets during optimization. Notably, adaptive (digital) interventions could introduce new resource considerations. Although AI-enabled interventions may be inexpensive to deliver, they may involve additional computational demands and implementation constraints that have not been typically considered an implementation constraint so far but may become part of policy frameworks in the future, for example, in relation to environmental impact or personal data. Considering MOST’s focus on explicit constraints, the framework is especially well suited to address these new challenges. Moreover, in a circular manner, such new developments can inform a re-specification of the optimization criterion, allowing the framework to evolve alongside the context in which it is applied. Taken together, the uptake of MOST has contributed to a wide use of factorial experiments as a rigorous and efficient tool for intervention optimization. However, MOST has so far often been applied with a primary focus on testing components within a fixed intervention with a factorial trial (phase 2), with key preparatory elements (phase 1) less consistently specified, limiting the translation of the results into clear optimization decisions (phase 2). At the same time, examples such as *myPlaybook* [60] and *Drink Less* [63] provide some preliminary evidence that when preparatory work is conducted more thoroughly and factorial trials are completed with richer data collection (eg, user experience, stakeholder input), even trials with null findings can still yield information that supports meaningful optimization. Considering these observations, we make several recommendations for future MOST applications (Textbox 1).

Textbox 1. **Recommendations for Multiphase Optimization Strategy (MOST) application to digital mental health interventions (DMHIs).**

1. Conduct and report the preparation phase transparently and in detail, using appropriate terminology. The goal is to establish a clear foundation for optimization rather than to refer to a general theoretical background.
  - Develop a fully specified conceptual model. Concretely, build a model in which components are independent from each other, and each targets a specific mechanism of change. Link these mechanisms to distal outcomes, operationalize them as proximal outcomes, and plan their measurement. Visualizing the model in a figure can greatly support clarity, operationalization, decision-making, and refinement [66].
  - Consider harvesting technological advancements such as AI that can be implemented with digital delivery to explore both effect modification and individual-level personalization.
  - Define an optimization criterion. Center the study’s objectives around optimization with respect to available resources rather than limiting the research question around the best combination of components alone. That is, define an optimization criterion early on, to avoid “default” retention of all components.
  - Conduct a pilot trial.
2. Plan the optimization phase to support informed component selection and iterative intervention development, recognizing that optimization may require multiple rounds of testing and refinement.
  - Plan to collect data necessary for resource-informed optimization and cumulative work. Concretely, measure resource indicators per component such as adherence, cost, completion duration, and clinical outcomes.
  - Consider what constitutes a meaningful effect size within your study conditions and define effect criteria, to help with the decision-making process once results from the trial are obtained.
  - Plan for multiple optimization rounds and return to the preparation phase if needed.

Several limitations should be acknowledged. First, studies were evaluated against expectations of a relatively comprehensive application of the MOST, which may not align with the authors’ original aims or MOST’s intended flexibility as a framework. Nonetheless, the review also showed that limited use of key preparatory principles may constrain optimization potential. Second, all judgments regarding activities within the preparation, optimization, and evaluation phases were based on the available papers. Apparent omissions may therefore reflect underreporting rather than absence, highlighting the need for clearer reporting of MOST-related decisions and processes, as also concluded in a previous review on the preparation phase [28]. Third, the MOST is a relatively recent framework, and studies completing all 3 phases are time- and resource-intensive. As a result, partial implementations are more prevalent in the literature, which may bias interpretations of how the MOST is currently applied. As shown in Table 2, there are more planned than completed optimization trials, further illustrating this early stage of application. The limited progression to the evaluation phase also makes it difficult to prove that optimized interventions have better outcomes than interventions developed and tested the standard way. In conclusion, by systematically assessing how the MOST has been applied so far and identifying the key barriers and opportunities in its use, this review lays essential groundwork for advancing a more rigorous, transparent, and cumulative approach to optimizing DMHIs. By combining a systematic search with structured data extraction across the MOST phases and qualitative synthesis of the completed key activities wherever needed, the review went beyond documenting the presence vs absence of specific elements to examine the role of MOST activities in scaffolding optimization. More broadly, the paper highlights a recurring challenge in psychological science: translating conceptual or therapeutic models into interventions that can be iteratively refined in a way that resembles the rigor of product optimization in engineering.

## Supplementary material

10.2196/98855 Multimedia Appendix 1 Search string.

10.2196/98855 Multimedia Appendix 2 Data extraction.

10.2196/98855 Multimedia Appendix 3 Risk of bias assessment.

10.2196/98855 Checklist 1 PRISMA-ScR checklist.

## Abbreviations

DMHI: digital mental health intervention
MOST: Multiphase Optimization Strategy
PRISMA-ScR: Preferred Reporting Items for Systematic Reviews and Meta-Analyses Extension for Scoping Reviews
RCT: randomized controlled trial
SMART: sequential multiple assignment randomized trial
