# Supplementary material for: Studies Using the Multiphase Optimization Strategy in Digital Mental Health: Scoping Review
Source: JMIR Ment Health. 2026 Nov 13;13:e98855. doi: 10.2196/98855 (PMC13766583; doi:10.2196/98855)
Supplement: Supplementary file 2 [file mental-v13-e98855-s001.docx]

Supplemental material - Search string

a. Search strategy: queries

Embase: 'multi-phase optimization strategy':ti,ab,kw OR 'multiphase optimization strategy':ti,ab,kw OR 'multi-phase optimization strategy':ti,ab,kw OR 'most framework':ti,ab,kw

CINAHL: AB “multi-phase optimization strategy” OR AB “multiphase optimization strategy” OR AB “multi-phase optimization strategy” OR AB “most framework”

PubMed: ((("multi-phase optimization strategy"[Title/Abstract]) OR ("multiphase optimization strategy"[Title/Abstract])) OR ("multi-phase optimization strategy"[Title/Abstract])) OR ("most framework"[Title/Abstract])

WOS: “multi-phase optimization strategy” (Topic) or “multiphase optimization strategy” (All Fields) or “multi-phase optimization strategy” (All Fields) or “most framework” (All Fields) and Article or Review Article (Document Types)

PsycInfo: (((tiab("multi-phase optimization strategy") OR tiab("multiphase optimization strategy") OR "multi-phase optimization strategy" OR "most framework") AND rtype.exact("Peer Reviewed Journal" OR "Journal Article" OR "Journal")) AND rtype.exact("Peer Reviewed Journal" OR "Journal Article" OR "Journal")) AND rtype.exact("Peer Reviewed Journal" OR "Journal Article" OR "Journal")

b. PRISMA 2020 Checklist
